# Homologous Recombination Related Signatures Predict Prognosis and Immunotherapy Response in Metastatic Urothelial Carcinoma

**DOI:** 10.3389/fgene.2022.875128

**Published:** 2022-04-26

**Authors:** Pan Li, Chaohu Chen, Jianpeng Li, Li Yang, Yuhan Wang, Zhilong Dong, Jun Mi, Yunxin Zhang, Juan Wang, Hanzhang Wang, Ronald Rodriguez, Junqiang Tian, Zhiping Wang

**Affiliations:** ^1^ Department of Urology, Lanzhou University Second Hospital, Lanzhou, China; ^2^ Key Laboratory of Gansu Province for Urological Diseases, Lanzhou, China; ^3^ Clinical Center of Gansu Province for Nephron-Urology, Lanzhou, China; ^4^ Department of Urology, University of Texas Health Science Center at San Antonio, San Antonio, TX, United States

**Keywords:** metastatic urothelial carcinoma, immune checkpoint inhibitors, homologous recombination, genomically unstable, predict prognosis

## Abstract

**Objective:** This study used homologous recombination (HR) related signatures to develop a clinical prediction model for screening immune checkpoint inhibitors (ICIs) advantaged populations and identify hub genes in advanced metastatic urothelial carcinoma.

**Methods:** The single-sample gene enrichment analysis and weighted gene co-expression network analysis were applied to identify modules associated with immune response and HR in IMvigor210 cohort samples. The principal component analysis was utilized to determine the differences in HR-related module gene signature scores across different tissue subtypes and clinical variables. Risk prediction models and nomograms were developed using differential gene expression analysis associated with HR scores, least absolute shrinkage and selection operator, and multivariate proportional hazards model regression. Additionally, hub genes were identified by analyzing the contribution of HR-related genes to principal components and overall survival analysis. Finally, clinical features from GSE133624, GSE13507, the TCGA, and other data sets were analyzed to validate the relationship between hub genes and tumor growth and mutation.

**Results:** The HR score was significantly higher in the complete/partial response group than in the stable/progressive disease group. The majority of genes associated with HR were discovered to be involved in the cell cycle and others. Genomically unstable, high tumor level, and high immune level samples all exhibited significantly higher HR score than other sample categories, and higher HR scores were related to improved survival following ICIs treatment. The risk scores for *AUNIP*, *SEPT*, *FAM72D*, *CAMKV*, *CXCL9*, and *FOXN4* were identified, and the training and verification groups had markedly different survival times. The risk score, tumor neoantigen burden, mismatch repair, and cell cycle regulation were discovered to be independent predictors of survival time following immunotherapy. Patients with a high level of expression of hub genes such as *EME1*, *RAD51AP1*, and *RAD54L* had a greater chance of surviving following immunotherapy. These genes are expressed at significantly higher levels in tumors, high-grade cancer, and invasive cancer than other categories, and are associated with TP53 and RB1 mutations.

**Conclusion:** HR-related genes are upregulated in genomically unstable samples, the survival time of mUC patients after treatment with ICIs can be predicted using a normogram model based on HR signature.

## Introduction

Urothelial carcinoma (UC) is the 10th most common type of cancer worldwide, predicted to cause 573,000 new cases and 213,000 deaths in 2020 alone (Sung et al., 2021). In industrialized countries, UC fatality rates have declined dramatically in recent years as a result of breakthroughs in prevention, early detection, and improved treatment (Babjuk et al., 2013). In the United States, the 5 years survival rate of patients with the early diagnosis is 95.8% because of multiple treatments such as surgery and bladder perfusion, while patients with metastatic urothelial carcinoma (mUC) is only 4.6% due to treatment limitations (Saginala et al., 2020). As a result, experts worldwide have been working nonstop to increase the survival rate of individuals with mUC.

Although platinum-based chemotherapy is the first-line treatment option for patients with mUC, cytotoxic drugs can cause clinical internal resistance, systemic toxicity, and other serious side effects, and approximately half of the patients should not be treated with platinum-based chemotherapy due to their age and pre-existing diseases (Klein and Hambley, 2009; Patel et al., 2020). Bacillus calmette guerin infusion, immune checkpoint inhibitors (ICIs), and other immunomodulatory therapy have all emerged as effective innovative therapeutics for mUC patients in recent years (Song et al., 2019). The United States Food and Drug Administration (FDA) has licensed several immunotherapy medicines (e.g., pembrolizumab, atezolizumab) for the first- or second-line treatment of individuals with mUC. While some individuals benefit from these treatments, between 70% and 80% of patients do not (Massard et al., 2016). In 2018, the FDA revised its license to require that ICIs be used exclusively in patients with mUC who do not respond to platinum-based chemotherapy but are positive for PD-L1 (Parikh et al., 2019). However, recent research indicates that the therapeutic benefit of PD-L1 is dependent on its expression on tumor-infiltrating immune cells, rather than on tumor cells (Mariathasan et al., 2018). At the same time, numerous technical issues limit the detection of PD-L1 expression, including the fact that it is easily missed in small biopsy samples, and the detection antibody’s PD-L1 binding is unstable (Topalian et al., 2016). As a result, the development of novel molecular markers capable of identifying prospective dominant populations is critical.

Tumor immunotherapy’s goal is to prevent tumor immune escape by inhibiting immunological checkpoints and to stimulate the immune system with neoantigens created by tumor-specific mutations (Hu et al., 2018). As a result, tumor mutation burden (TMB) and tumor neoantigen burden (TNB) can be utilized to predict the efficacy of ICIs (Chan et al., 2019; Wang et al., 2021). A recent study has revealed that the DNA damage repair (DDR) pathway is not only connected with platinum chemotherapeutic drug susceptibility, but also with tumor mutation. As a result of these alterations, genomically instability, neoantigen generation, and upregulation of PD-L1 expression occur (Jiang et al., 2021). Additionally, Park et al. (2019) found that abnormal mutations in both the double-stranded and single-stranded DNA damage repair pathways are related to TMB in patients with small-cell lung cancer. Homologous recombination (HR) contributes to gene stability in two ways, it is a highly accurate repair process that maintains genomic stability when double-stranded DNA is damaged in normal cells, but it can result in genomic instability when hyperactive. For example, Shammas et al. (2009) discovered that HR is particularly active in myeloma and that inhibiting them considerably reduces genomically instability. As a consequence, studying HR-mediated immune responses and tumor alterations are essential.

In this study, the relationship between different DDR pathways and immunotherapy response was deeply analyzed, and a clinical prognosis model based on the HR signature was constructed. This model can be used to predict the prognosis of mUC patients following immunotherapy and to identify potential hub genes, paving the path for more basic experimental studies.

## Data and Methods

### Data Sources

**FIGURE 1:**
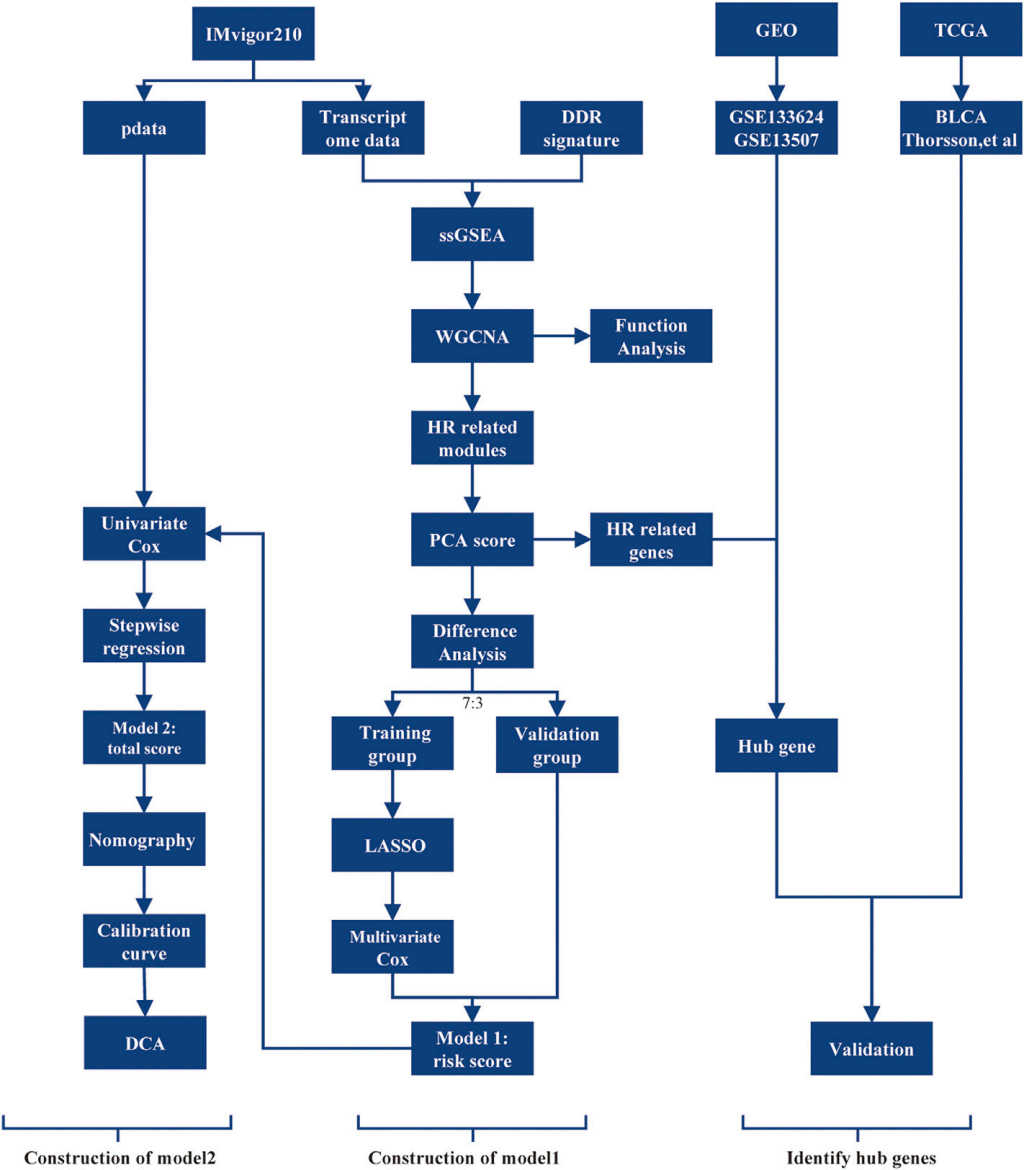
The workflow of the selection process for the eligible studies in the analysis. DDR, DNA damage repair; ssGSEA, single sample gene set enrichment analysis; WGCNA, weighted gene co-expression network analysis; HR, homologous recombination; PCA, principal component analysis; LASSO, least absolute shrinkage, and selection operator; DCA, decision curve analysis; TCGA, The Cancer Genome Atlas; GEO, Gene Expression Omnibus.

Clinical characteristics and tumor tissue transcriptome counts for mUC patients were collected from the IMvigor210 cohort (http://research-pub.gene.com/IMvigor210CoreBiologies/packageVersions/) (Mariathasan et al., 2018), a phase 2 clinical investigation of atezolizumab in advanced mUC. The study removed samples from patients whose treatment outcomes were unknown. 298 samples were ultimately obtained. Additionally, the study obtained the expression profiles of 165 primary bladder cancer samples and 21 paired tissue samples from the GSE13507 and GSE133624 in GEO database (https://www.ncbi.nlm.nih.gov/geo/), and the TCGA BLCA transcriptome counts sequencing data and sample characteristic data from UCSC Xena (http://xena.ucsc.edu/) and Thorsson et al. (2018). The study obtained 397 tumor transcriptome data, removing samples with no discernible immunological subtype and several samples from the same patient. For future analysis, RNAseq transcriptome data were transformed to transcripts per million the data for the chip expression profile data were normalized using the “limma” package’s functions. The workflow was displayed in Figure 1.

### Single Sample Gene Set Enrichment Analysis

ssGSEA is a nonparametric method for normalizing the gene expression values in a given sample and generating enrichment scores based on the empirical cumulative distribution functions of the signature genes and the remaining genes (Barbie et al., 2009). Ten gene sets representing distinct DDR pathways were retrieved from the literature (Kang et al., 2012) and ssGSEA analysis was conducted using the “GSVA” package in R4.1.1 (R Foundation for Statistical Computing, Vienna, Austria) (Hänzelmann et al., 2013). Then, the study examined the association between the DDR pathway enrichment score and immunological response.

### Weighted Gene Co-Expression Network Analysis

The purpose of WGCNA is to identify co-expressed gene modules and to investigate the relationship between gene networks and the targeted phenotypes, as well as the network’s hub genes (Zhang and Horvath, 2005). According to the WGCNA official guidance document (https://horvath.genetics.ucla.edu/html/CoexpressionNetwork/Rpackages/WGCNA/), the study evaluated the top 5000 genes according to the median absolute deviation for WGCNA and associated immunotherapy response, enrichment score of DDR-related pathways, and other features using the “WGCNA” package. Modules with a cluster height less than 0.25 are merged to eliminate duplicates (Langfelder and Horvath, 2008).

### Function Analysis

Gene ontology (GO) analysis calculates *p* values for provided gene sets in biological processes, molecular functions, cellular components, and major pathways using the hypergeometric distribution approach. Determine whether the number of genes in a particular gene set surpasses the number of genes that would be expected randomly in terms of function or route. Enrichment of gene ontology analysis of genes inside each module using the anRichment program (https://horvath.genomics.ucla.edu/html/CoexpressionNetwork/GeneAnnotation/). The three most significant components were eliminated based on the *p*-value.

### Principal Component Analysis

Principal component analysis (PCA) is a technique for reducing high-dimensional data sets to low-dimensional data sets. Its scores are weighted according to the importance of each gene in the first principal component, resulting in a weighted average that can be used to assess the biological functions of signature genes (Berglund et al., 2017). The “IOBR” package’s PCA method was utilized to perform feature scoring on samples based on HR-related modules (Zeng et al., 2021). The tissue subtypes of the samples in the research cohort were determined using the “BLCAsubtyping” package (Kamoun et al., 2020). The study investigated the distribution of HR scores among subtypes, tumor level, immune level, and overall survival.

### Difference Analysis

The “Maxstat” package was used to determine the optimal survival threshold for HR scores from IMvigor210 cohort samples divided into high and low expression groups (Lausen and Schumacher, 1992). The differentially expressed gene (DEGs) screening standard used to develop the clinical prognosis model of tumor immunotherapy was the use of the “Desq2” package (Love et al., 2014), filter genes count expresses overall less than 10, log2 |Fold Change |>1 and p.adj<0.05 were used to identify DEGs.

### Construction of Risk Prediction Model

In a 7:3 ratio, the IMvigor210 cohort was divided into training and validation groups. In the training group, the least absolute shrinkage and selection operator (LASSO) COX regression model was generated using pairs of differential genes from the “glmnet” package, and the lambda value with the lowest value was chosen as the optimal lambda value (Simon et al., 2011). The selected variables were included in the multivariate COX regression model, and variables that did not support the proportional hazards hypothesis were eliminated from the second regression. The “rms” package was used to calculate the variance inflation factor of each variable and assess whether the model’s variables’ expressions were multicollinear, and ultimately, derive the risk variables and risk coefficients.

$$\mathrm{Risk}\;\text{score=}\overset{n}{\underset{\mathrm{i=1}}{\sum }}\left(\mathrm{Expression}\;\mathrm{mRNA}i\;\;\mathrm{\times Coefficient}\;\mathrm{mRNA}i\right)$$

The samples were separated into high and low risk groups based on their median risk score, and KM survival analysis was performed on the training and verification groups.

### Construction and Validation of a Prognostic Nomogram

On the risk score and sample feature score, a univariate COX regression analysis was conducted. The standard screening variable was *p* < 0.05. Following the exclusion of samples with missing variables. The stepwise regression method (https://cran.r-project.org/web/packages/My.stepwise/index.html) was utilized to filter covariates, and a multivariate COX regression prognostic model was created. The variance inflation factor threshold was adjusted at 4 to avoid multicollinearity between variables. Nomogram was developed to investigate the association between total score and one- and two-year prognoses. Finally, the calibration curve and decision curve are used to assess the model’s predictive ability.

### Screening of Important Homologous Recombination Genes

The first principal component was discovered, and the primary HR related genes contributing to it were identified using the expected average contribution value (1/length (variables)*100%) as a criterion. Using the median gene expression value as the crucial value, samples from the IMvigor210 cohort were separated into high and low expression groups, and the log-rank test was performed to examine the difference between the expression level and the immunotherapy prognosis level. To ensure that *p* < 0.05 was utilized as the screening threshold for module hub genes.

### Verify the Relationship Between Hub Genes and Clinical Features

Transcriptome sequencing data of bladder cancer and paired paracancer samples from GSE133624 were used to analyze the expression of hub genes in tumor samples and paired normal samples. GSE13507 array expression profile data were used to analyze the differences of hub genes in different pathological grades and aggressiveness, as well as their relationship with specific survival of patients.

### Analysis of Hub Genes and Tumor Progression Mechanism

Thorsson et al. (2018) scored the number of tumors in the TCGA database according to the characteristics of different biofunctional molecules. Pearson correlation analysis was conducted on hub gene expression levels and characteristic scores using the “Psych” package, and correlation heat maps were drawn. In order to cor > 0.7 and *p* < 0.001 as a strong correlation screening criterion. Based on the mutation data in the immunotherapy cohort, the differences of hub genes in different mutated and non-mutated samples were analyzed, and their relationships with TMB and TNB were analyzed.

### Statistical Analysis

The statistics in this study were based on the software package in R4.1.1. The difference analysis of two samples for quantitative data conforming to normal distribution adopts independent sample t-test, the non-parametric test was adopted for non-normal distribution quantitative data, the non-parametric test was adopted for various quantitative data, Chi-square test was adopted for counting data, and the log-rank test was adopted for Kaplan-Meier analysis. Correlation analysis was conducted by Pearson analysis. Take *p* < 0.05 was the standard of statistical significance.

## Results

### The Homologous Recombination Enrichment Score was Considerably Greater in the Complete/Partial Response Group Than in the Stable/Progressive Disease Group

**FIGURE 2:**
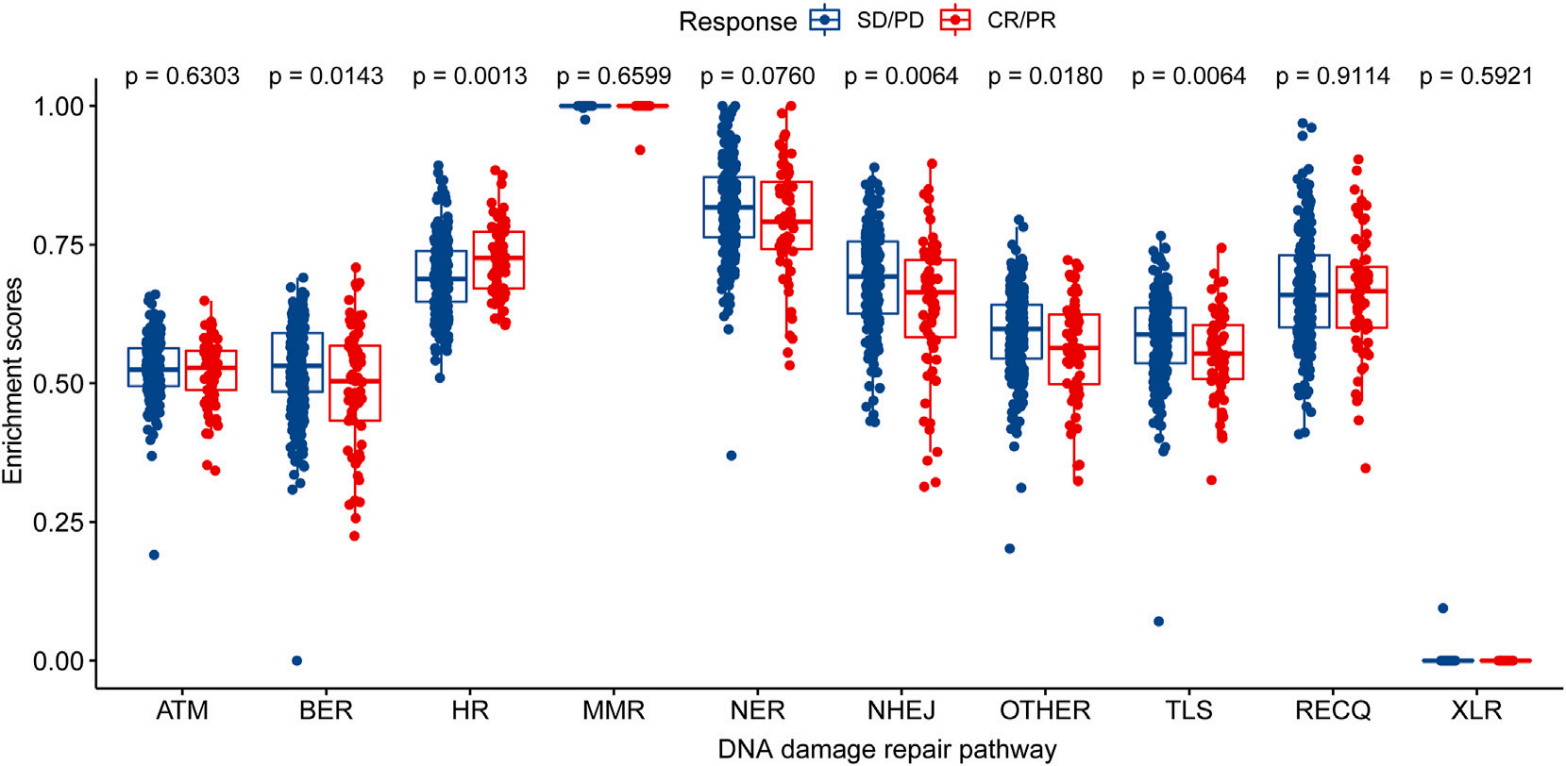
Distribution of enrichment scores of DNA damage repair pathway in CR/PR group and SD/PD group. ATM, ataxia telangiectasia mutated; BER, base excision repair; FA/HR, Fanconi Anemia/homologous recombination; MMR, mismatch repair; NER, nucleotide excision repair; NHEJ, non-homologous end joining; OTHER, other; TLS, translesion synthesis; RECQ, recQ helicase pathway; XLR, cross-link repair; CR/PR, complete response/partial response; SD/PD, stable disease/progressive disease.

The enrichment analysis of ten different DNA damage repair pathways revealed that the HR pathway had a considerably higher enrichment score in the CR/PR group than in the SD/PD group (*p* = 0.00086). Base excision repair (BER), non-homologous end joining (NHEJ), translesion synthesis (TLS), and other scores were considerably lower in the CR/PR group than in the SD/PD group (*p* < 0.05), demonstrating that the genes involved to HR were associated with immunotherapy result (Figure 2).

### Recognize Modules Involved in Immunotherapy Response and DNA Damage Repair

**FIGURE 3:**
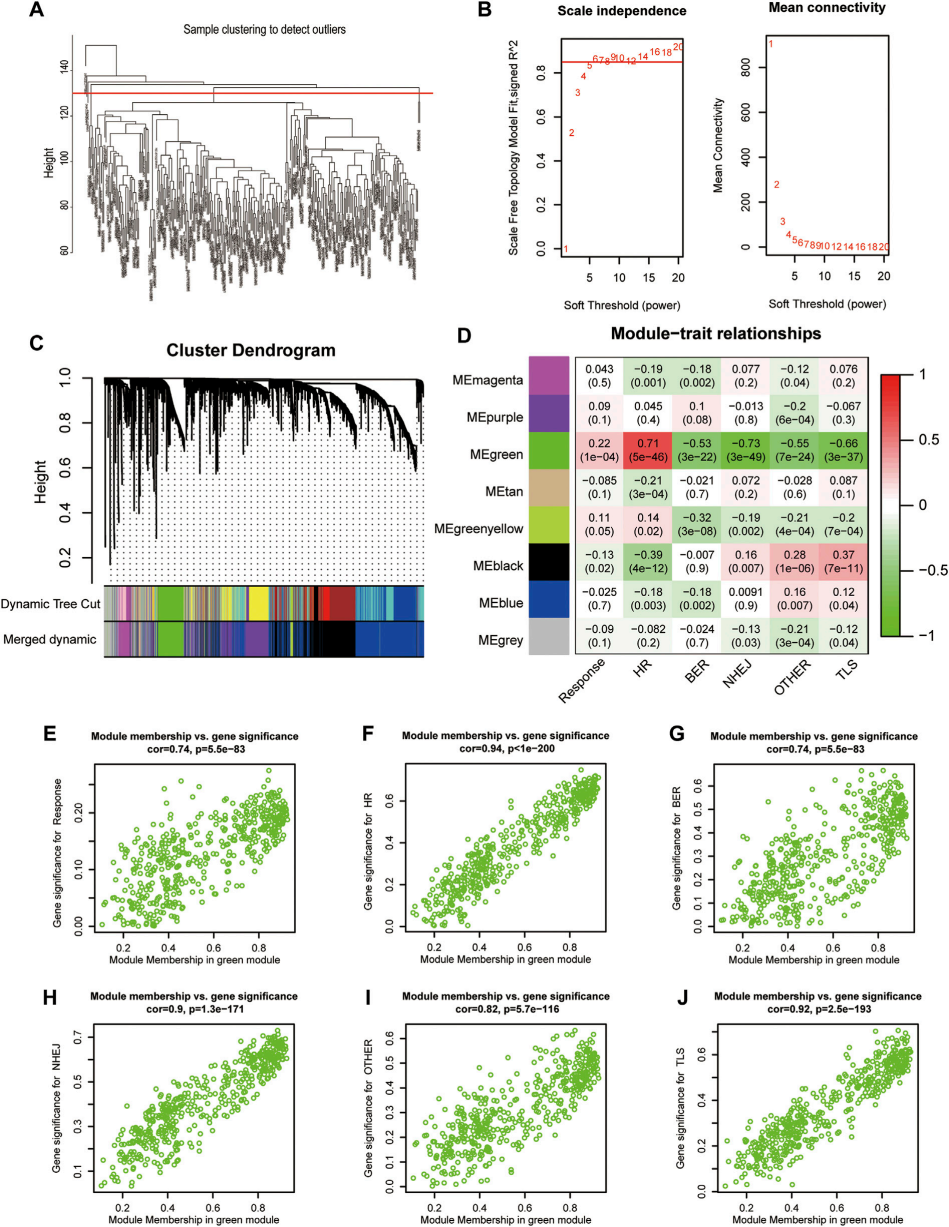
Construction of gene weighted co-expression network. **(A)**, Sample cluster diagram. **(B)**, Analysis of the scale-free topology model fit index for soft threshold powers (β). **(C)**, A cluster dendrogram was built based on the dissimilarity of the topological overlap, which presents different gene co-expression modules in IMvigor210, modules with a clustering degree less than 0.25 were merged. **(D)**, Heatmap analysis of the correlation between module eigengenes of IMvigor210 and enrichment score of DNA damage repair pathway. **(E–J)**, The scatterplot of GS for the response, different enrichment score of DNA damage repair pathway vs. MM in the green module. FA/HR, Fanconi Anemia/homologous recombination; BER, base excision repair; NHEJ, non-homologous end joining; OTHER, other; TLS, translesion synthesis; GS, gene significance; MM, module membership.

Immunotherapy response data, as well as HR, BER, NHEJ, OTHER, and TLS signature enrichment scores, were used to construct gene co-expression networks containing the top 5000 MDA-valued genes. The study removed samples having a cluster height of more than 130, as they were considered outliers. When the network’s soft threshold is set to 6, it is possible to design a co-expression network. These genes can be classified into eight modules according to their expression (Figures 3A–C). These modules include magenta, purple, green, tan, greenyellow, black, blue, and grey. Correlations between the green module gene co-expression network and treatment response (cor = 0.22, *p* = 1E-4) and HR (cor = 0.71, *p* = 5E-46) were significantly positive. Correlations with BER (cor = −0.53, *p* = 3E-22), NHEJ (cor = −0.73, *p* = 3E-49), OTHER (cor = −0.55, *p* = 7E-24), and TLS (cor = −0.66, *p* = 3E-37) were negative (Figure 3D). The module membership (MM) and treatment response (cor = 0.74, *p* = 5.5E-83), HR (cor = 0.94, *p* = 1E-200), BER (cor = 0.74, *p* = 5.5E-83), NHEJ (cor = 0.9, *p* = 1.3E-171), OTHER (cor = 0.82, *p* = 5.7E-116), TLS (cor = 0.92, *p* = 2.5E-193), and OTHER gene signatures were significantly correlated (Figures 3E–J). Finally, the green module’s 472 genes were used to generate the HR-related gene signature of mUC patients.

### Module-Level Study of Gene Ontology Functions

The WGCNA modules were analyzed using the GO function analysis, and the results indicated that green modules with a significant positive correlation with immunotherapy response and a high HR enrichment score were primarily enriched in the cell cycle (GO.BP: 0007049), cell cycle process (GO.BP: 0022402), and mitotic cell cycle (GO.BP: 0000278). Negatively correlated black modules included exterior encapsulating structure (GO.CC: 0030312), extracellular matrix (GO.CC: 0031012), collagen-containing extracellular matrix (GO.CC: 0062023), and others. Immunological response (GO.BP: 0006955), immune system process (GO.BP: 0002376), leukocyte activation (GO.BP: 0045321), and other immune reaction-related GO items were primarily enriched in the blue module (Table 1).

**TABLE 1:** GO enrichment analysis of modules in WGCNA (Top3).

Module	Rank	ID	Type	Description	Bonferroni
Black	1	GO:0030312	CC	External encapsulating structure	1.07E-39
	2	GO:0031012	CC	Extracellular matrix	1.07E-39
	3	GO:0062023	CC	Collagen-containing extracellular matrix	1.52E-37
Blue	1	GO:0006955	BP	Immune response	1.18E-61
	2	GO:0002376	BP	Immune system process	7.81E-52
	3	GO:0045321	BP	Leukocyte activation	1.39E-48
Green	1	GO:0007049	BP	Cell cycle	7.30E-79
	2	GO:0022402	BP	Cell cycle process	3.04E-73
	3	GO:0000278	BP	Mitotic cell cycle	9.44E-70
Green-yellow	1	GO:0051607	BP	Defense response to virus	5.12E-29
	2	GO:0140546	BP	Defense response to symbiont	5.12E-29
	3	GO:0034340	BP	Response to type I interferon	2.67E-27
Magenta	1	GO:0006396	BP	RNA processing	7.19E-250
	2	GO:0005730	CC	Nucleolus	4.12E-190
	3	GO:0016070	BP	RNA metabolic process	1.95E-96
Purple	1	GO:0000977	MF	RNA polymerase II transcription regulatory region sequence-specific DNA binding	3.92E-05
	2	GO:0003700	MF	DNA-binding transcription factor activity	1.52E-04
	3	GO:0000976	MF	Transcription regulatory region sequence-specific DNA binding	4.00E-04
Tan	1	GO:0007156	BP	Homophilic cell adhesion via plasma membrane adhesion molecules	7.72E-42
	2	GO:0098742	BP	Cell-cell adhesion via plasma-membrane adhesion molecules	1.05E-35
	3	GO:0005509	MF	Calcium ion binding	2.22E-24

GO, gene ontology; WGCNA, weighted gene co-expression network analysis; BP, biological process; CC, cellular component; MF, molecular function.

### The Homologous Recombination Scores of Several Feature Samples Varied Significantly

**FIGURE 4:**
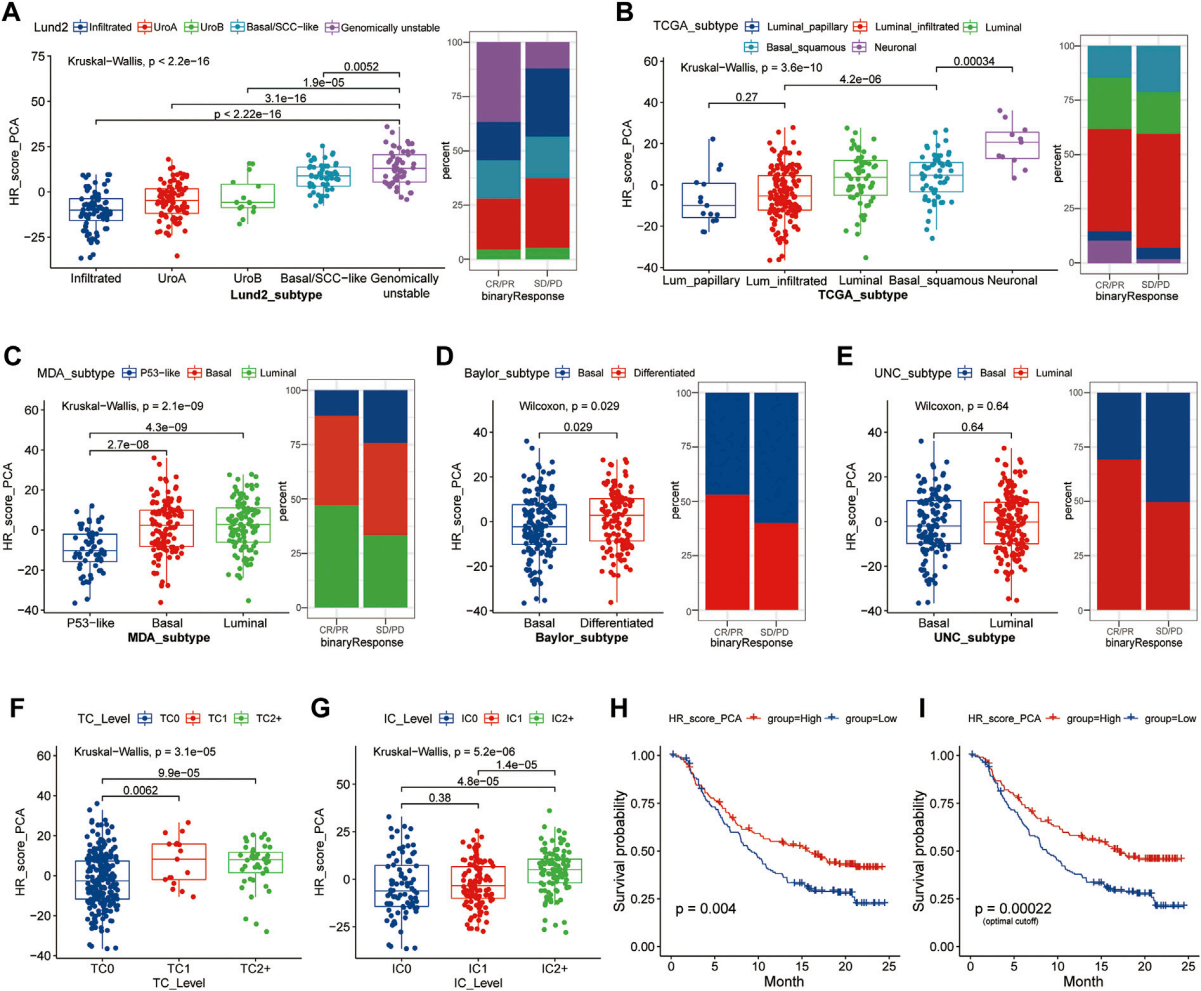
HR score distribution in different subtypes of samples based on different molecular features. **(A–E)**, The distribution of HR scores in different subtypes was based on Lund2, TCGA, MDA, Baylor, and UNC classifications methods, as well as the composition ratio of different subtypes in the CR/SP and SD/PD groups; **(F, G)**, The distribution difference of HR score in different tumor level and immune level; **(H,I)**, Kaplan-Meier survival analysis was performed on patients in the high and low groups according to the median HR expression value **(H)** or optimal HR cut-off value **(I)**. Lund, Lund University; TCGA, The Cancer Genome Atlas; MDA, MD Anderson Cancer Center, UNC, University of North Carolina; HR, homologous recombination; TC, tumor cells; IC, immune cells; CR/PR, complete response/partial response; SD/PD, stable disease/progressive disease.

The signatures of HR-related modules were scored using PCA. The results indicated that the HR scores of the Lund2, TCGA, MDA, and Baylor subtypes were substantially different (*p* < 0.05) (Figures 4A–D). The Lund2 subtype had a considerably higher score of genomically instability than the other subtypes, and the sample composition score of genomically instability in the CR/PR group (36.76%) was significantly greater than that in the SD/PD group (12.17%). Neuronal (TCGA), luminal (MDA), and differentiated (Baylor) subtypes had significantly higher HR values than the other subtypes (*p* < 0.05). The HR scores for high tumor level (TC2+) and high immune level (IC2+) were substantially higher than those for low tumor level (TC0) and low immunity level (IC0) (*p* < 0.05) (Figures 4F,G). The overall survival rate of patients with a high score was substantially greater than that of patients with a low score (*p* < 0.05) (Figures 4H,I).

### Clinical Features and Functional Gene Expression Were Different Across Groups With High and Low Homologous Recombination Score

**FIGURE 5:**
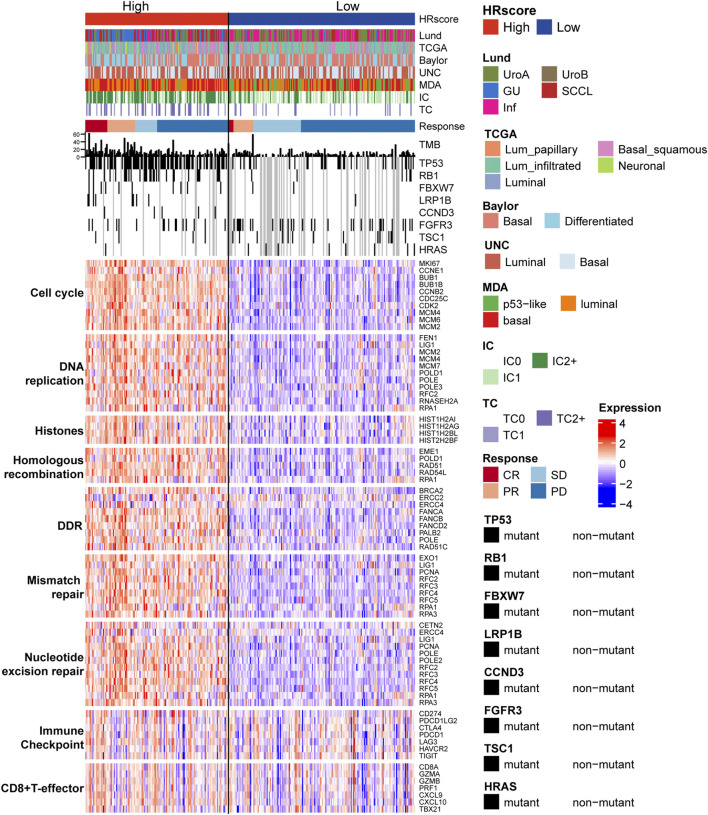
The relationship between HR score groups, different subtypes, and core biological pathways. Rows of the heat map show gene expression (z-scores) grouped by pathway. Lund, Lund University; TCGA, The Cancer Genome Atlas; MDA, MD Anderson Cancer Center, UNC, University of North Carolina; HR, homologous recombination; TC, tumor cells; IC, immune cells; CR, complete response; PR, partial response; SD, stable disease; PD, progressive disease; DDR, DNA damage repair.

The study divided the samples into groups according to the ideal survival threshold for HR score and discovered that when the critical HR score was 2.34, the overall survival Kaplan-Meier analysis of the high and low score groups was the most significant (*p* < 0.001) (Figures 4–I). There were significant differences in treatment response, tumor level, immune level, TCGA subtype, and immunophenotype between the two groups (*p* < 0.05), but not in gender, intravesical BCG infusion, smoking history, metastatic site, or other features (*p* > 0.05) (Table 2). The comparison of high and low HR score samples revealed that TMB and the number of mutations in TP53, RB1, FBXW7, LRP1B, CCND3, FGFR3, TSC1, and HRAS were considerably higher in the high score group than in the low score group. Additionally, genes are involved in biological processes such as the cell cycle, DNA replication, histones, HR, DDR, mismatch repair, nucleotide excision repair, immune checkpoint, and CD8+ T-effector had significant differences between the high and low groups (Figure 5).

**TABLE 2:** Patient characteristics.

Characteristic	Overall, N = 298^1^	HR score group		*p* Value^2^
		High, *n* = 129^1^	Low, *n* = 169^1^	
Sex				0.2
Female	65 (22%)	33 (26%)	32 (19%)	
Male	233 (78%)	96 (74%)	137 (81%)	
Response				<0.001
PR	43 (14%)	25 (19%)	18 (11%)	
CR	25 (8.4%)	20 (16%)	5 (3.0%)	
PD	167 (56%)	64 (50%)	103 (61%)	
SD	63 (21%)	20 (16%)	43 (25%)	
IC Level				<0.001
IC0	83 (28%)	26 (20%)	57 (34%)	
IC1	112 (38%)	35 (27%)	77 (46%)	
IC2+	102 (34%)	68 (53%)	34 (20%)	
Unknown	1	0	1	
TC Level				<0.001
TC0	238 (80%)	89 (69%)	149 (89%)	
TC1	17 (5.7%)	9 (7.0%)	8 (4.8%)	
TC2+	42 (14%)	31 (24%)	11 (6.5%)	
Unknown	1	0	1	
TCGA subtype				<0.001
I	107 (36%)	27 (21%)	80 (47%)	
II	75 (25%)	46 (36%)	29 (17%)	
III	60 (20%)	27 (21%)	33 (20%)	
IV	56 (19%)	29 (22%)	27 (16%)	
Immune phenotype				0.002
Desert	69 (28%)	25 (23%)	44 (33%)	
Excluded	113 (46%)	45 (41%)	68 (51%)	
Inflamed	62 (25%)	40 (36%)	22 (16%)	
Unknown	54	19	35	
Intravesical BCG				0.4
No	231 (78%)	103 (80%)	128 (76%)	
Yes	67 (22%)	26 (20%)	41 (24%)	
Tobacco History				0.8
Current	32 (11%)	15 (12%)	17 (10%)	
Never	98 (33%)	40 (31%)	58 (34%)	
Previous	168 (56%)	74 (57%)	94 (56%)	
Met Disease Status				0.3
Liver	81 (30%)	33 (29%)	48 (30%)	
LN Only	51 (19%)	26 (23%)	25 (16%)	
Visceral	139 (51%)	54 (48%)	85 (54%)	
Unknown	27	16	11	

1 n (%).

2 Pearson’s Chi-squared test; Fisher’s exact test.

HR, homologous recombination; CR, complete response; PR, partial response; SD, stable disease; PD, progressive disease; TCGA, the cancer genome atlas; BCG: bacillus calmette guerin vaccine; IC, immune cells; TC, tumor cells.

### Identifying Prognostic Genes and Developing and Validating Risk Prognostic Models

**FIGURE 6:**
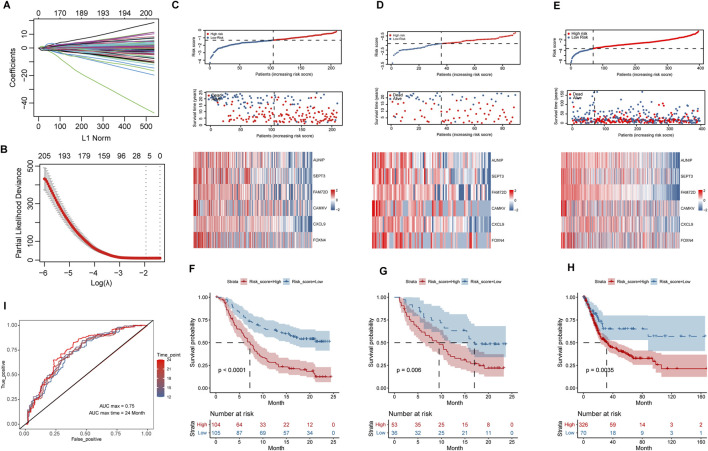
Construction and validation of survival risk model after immunotherapy. **(A,B)**, Variables were filtered using LASSO regression, according to DEGs. **(C–E)**, Distribution of risk scores, survival status, and heat maps of hub variables in IMvigor210 training group and internal validation group, as well as TCGA_BLCA cohort. **(F–H)**, Kaplan-Meier survival analysis of high and low risk groups in IMvigor210 training group and internal validation group, and TCGA_BLCA cohort. **(I)**, Time-dependent ROC curves based on risk scores in the IMvigor210 cohort.

The differential analysis of high and low group samples yielded a total of 1512 DEGs, including 797 upregulated and 715 downregulated genes. In the training group, LASSO regression was done with a lambda of 0.1422 (Figures 6A,B). Six HR-related immunotherapy prognostic genes were examined, and the risk scoring formula was derived using multivariate COX regression: Risk score = AUNIP* (−0.09913798) + SEPT3* (−0.11228204) + FAM72D* (−1.03789188) + CAMKV* (−1.05763747) + CXCL9* (−0.15162375) + FOXN4* (−0.32027958). Kaplan-Meier survival analysis using the training group’s median risk score as the threshold revealed that high-risk patients’ overall survival time was considerably shorter than that of low-risk patients in the training and validation groups (*p* < 0.01) (Figures 6C,D,F,G). Overall survival was likewise significantly worse in high-risk patients than in low-risk patients in the TCGA BLCA cohort (without immunotherapy) at the optimum threshold (*p* < 0.01) (Figures 6E,H). To assess the model’s diagnostic value, a time-dependent ROC analysis was performed; the findings indicated that the AUC Max value was 0.75, indicating that the model had a high predictive value for the prognosis of patients undergoing immunotherapy (Figure 6I).

### Develop a Nomogram for Survival Prediction

**FIGURE 7:**
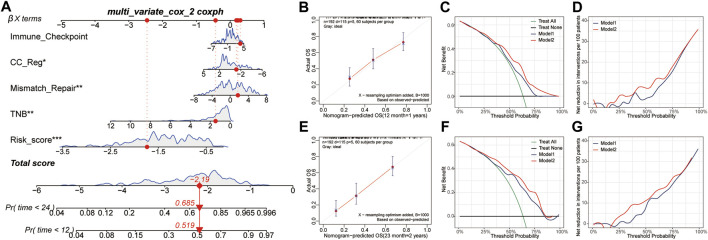
Construction and validation of survival risk model after immunotherapy. **(A)**, The Nomogram is composed of risk score and signature score of core biological function. **(B,E)**, Calibration curves of patients’ 1-year and 2-year survival after immunotherapy based on the total score model. **(C,D,F,G)**, The decision curve and net benefit curve of patients’ survival at 1 and 2 years after immunotherapy, according to different models. CC reg, cell cycle regulation; TNB, tumor neoantigen burden.

COX analysis was done univariately on the risk score and feature scores of the samples’ various functions, and 19 factors associated with overall survival were screened, as shown in Table 3. Variables were screened using stepwise regression, and multivariate COX regression analysis was used to generate a column chart for five variables: risk score, TNB, mismatch repair, CC Reg, and immunological checkpoint (Figure 7A). Among them, risk score and mismatch repair were independent risk factors for overall patient survival, whereas TNB and CC Reg were independent protective factors, as shown in Table 4. The calibration curve and decision curve were used to assess the total score model’s predictive value for patient survival time 1 and 2 years after immunotherapy, and the results indicated that the predicted total survival time was highly consistent with patient survival time in practice (Figures 7B,E). Patients benefited from the use of both models, and the benefit degree of model 2 (total score) was greater than that of model 1 (risk score) (Figures 7C,D,F,G).

**TABLE 3:** Univariate Cox regression analysis.

Characteristic	Beta	Hazard ratio (95% CI for HR)	*p* Value	
Sex	−0.17	0.84 (0.6–1.2)	3.10E-01	
IC level	−0.34	0.71 (0.59–0.85)	2.10E-04	***
TC level	−0.03	0.98 (0.8–1.2)	8.10E-01	
TMB	−0.05	0.96 (0.93–0.98)	3.10E-04	***
TNB	−0.36	0.7 (0.59–0.83)	6.10E-05	***
Lund2	0.19	1.2 (1.1–1.4)	1.70E-03	**
TCGA subtype	0.00	1 (0.88–1.1)	9.60E-01	
HR score group	−0.56	0.57 (0.42–0.77)	2.60E-04	***
Immune checkpoint	−0.08	0.93 (0.87–0.99)	1.50E-02	*
CD 8 T effector	−0.10	0.91 (0.86–0.96)	3.30E-04	***
DDR	−0.04	0.96 (0.94–0.99)	4.80E-03	**
APM	−0.07	0.93 (0.87–1)	4.50E-02	*
CC Reg	0.10	1.1 (1–1.2)	1.80E-02	*
Fanconi	−0.07	0.94 (0.9–0.97)	7.80E-04	***
Gene19	0.03	1 (0.98–1.1)	2.40E-01	
Tcga	0.01	1 (0.94–1.1)	8.30E-01	
Histones	−0.08	0.92 (0.85–1)	5.00E-02	
EMT1	0.02	1 (0.95–1.1)	6.30E-01	
EMT2	0.03	1 (0.96–1.1)	4.00E-01	
EMT3	0.01	1 (0.94–1.1)	7.40E-01	
WNT target	0.17	1.2 (1–1.4)	8.30E-03	**
FGFR3 related	0.05	1.1 (0.95–1.2)	2.90E-01	
Cell cycle	−0.03	0.97 (0.95–0.99)	1.30E-02	*
Mismatch repair	−0.06	0.95 (0.9–0.99)	2.70E-02	*
Homologous recombination	−0.08	0.93 (0.88–0.98)	3.40E-03	**
Nucleotide excision repair	−0.05	0.95 (0.91–1)	3.20E-02	*
DNA replication	−0.04	0.96 (0.92–0.99)	1.50E-02	*
Base excision repair	−0.05	0.95 (0.9–1)	4.20E-02	*
Risk score	0.82	2.3 (1.8–2.9)	5.70E-11	***

***p < 0.001; **p < 0.01; *p < 0.05.

TMB, tumor mutation burden; TNB, tumor neoantigen burden; IC, immune cells; TC, tumor cells; HR, homologous recombination; DDR, DNA, damage repair; APM: antigen processing and presentation machinery; CC, reg, cell cycle regulation.

**TABLE 4:** Multivariate Cox regression analysis.

Characteristic	Beta	Hazard ratio (95% CI for HR)	*p* Value	
Risk score	1.42	4.1 (2.4–7.1)	4.10E-07	***
TNB	−0.30	0.74 (0.61–0.91)	0.0039	**
Mismatch Repair	0.13	1.1 (1–1.3)	0.0033	**
CC Reg	−0.16	0.85 (0.74–0.98)	0.03	*
Immune Checkpoint	0.08	1.1 (0.97–1.2)	0.12	

Global *p*-value (Log-Rank): 4.5977E-11; AIC: 1059.89; Concordance Index: 0.7.

***p < 0.001; **p < 0.01; *p < 0.05.

TNB, tumor neoantigen burden; CC, reg, cell cycle regulation.

### Identify Critical Homologous Recombination-Related Genes Inside the Module

**FIGURE 8:**
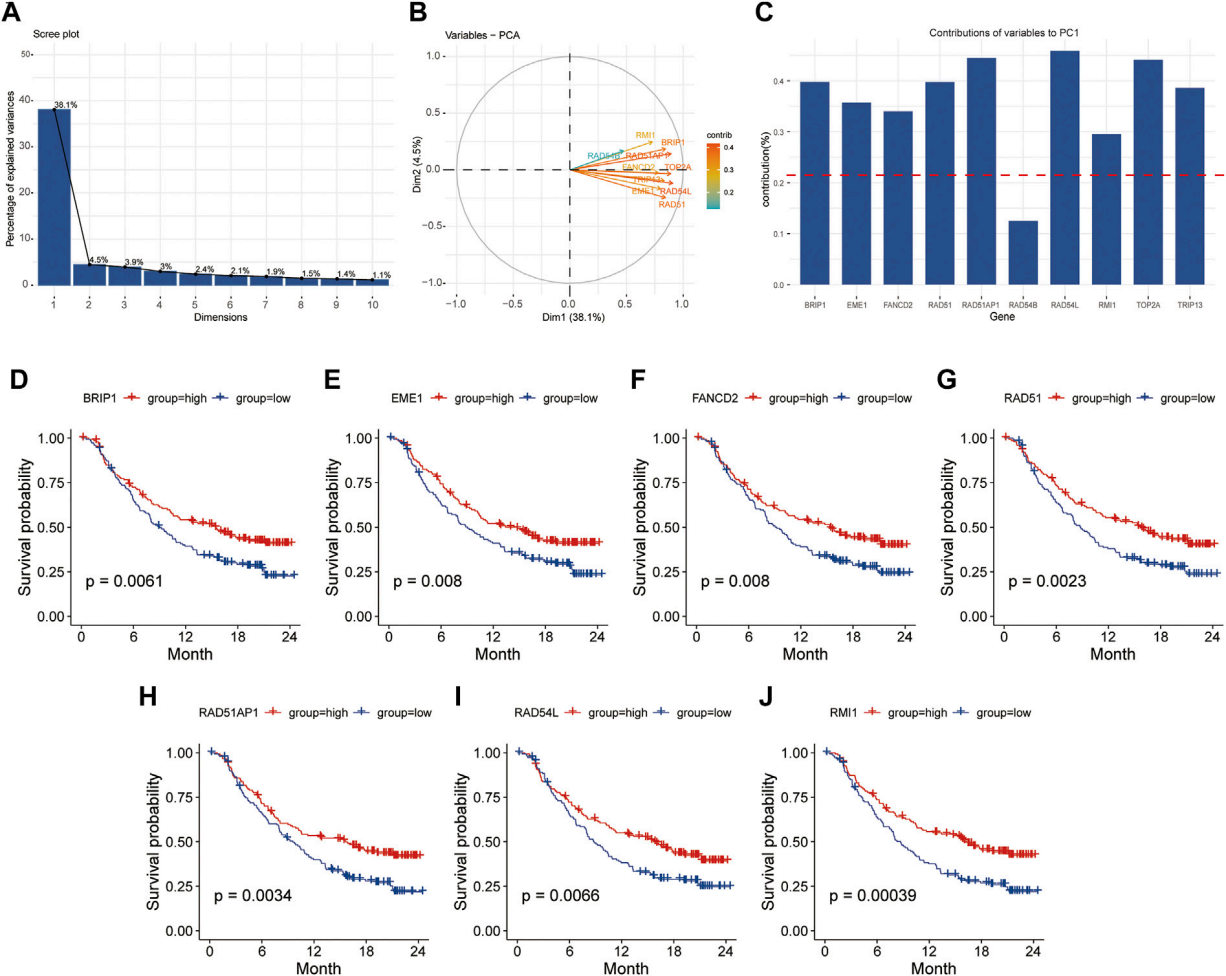
The first principal component of PCA was used to screen for HR-related genes. **(A)**, Explainability of different principal components to all variables; **(B, C)**, Contribution of HR related genes to the first principal component. **(D–J)**, Kaplan-Meier survival analysis based on grouping of median expression values of HR related genes. PCA, principal component analysis; HR, homologous recombination.

PCA was used to find the hub genes affecting HR scores. The proportion of explanatory variables in the first principal component (38.1%) was significantly greater than that in the other (Figure 8A). The intersection of the genes in the first component and the HR signature (GO: 0035825) genes in the GO database revealed that nine genes provided more than the average contribution to the ten genes obviously implicated in HR (Figures 8B,C). Additionally, survival analysis revealed that patients in the high expression group of *BRIP1*, *EME1*, *FANCD2*, *RAD51*, *RAD51AP1*, *RAD54L*, and *RMI1* genes had a significantly longer overall survival time than those in the low expression group prior to immunotherapy (*p* < 0.05), and patients had a better prognosis following immunotherapy (*p* < 0.05) (Figures 8D–J).

### Signature Gene Expression was Found to be Associated With Carcinogenesis and Progression

**FIGURE 9:**
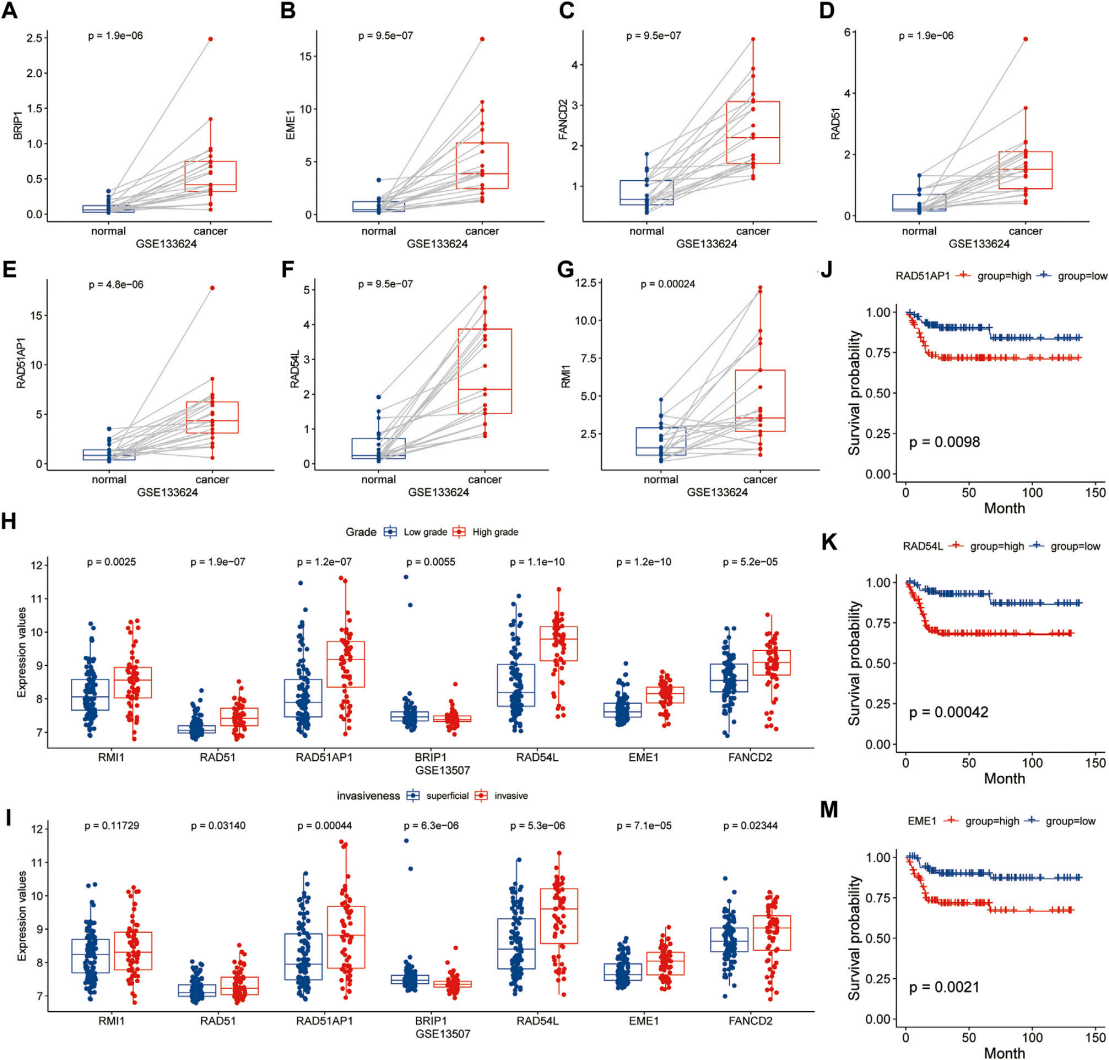
Analysis of relationship between HR related genes and clinical features in GSE133624 and GSE13507. **(A–G)**, Differences in expression of HR-related genes between cancer and paired paracancer samples; **(H,I)**, Expression differences of HR related genes in different tumor grades and invasive samples; **(J–M)**, Kaplan-Meier survival analysis based on grouping of median expression values of HR related genes in GSE13507.

Further examination of the relationship between signature genes and tumor occurrence and progression revealed that in patients without immunotherapy, the expression levels of seven signature genes were significantly higher in tumor tissues than in para-cancer tissues (*p* < 0.05) (Figures 9A–G). The expression levels of *EME1*, *RAD51*, *RAD51AP1*, and *RAD54L* genes were significantly higher in high grade bladder cancer and invasive bladder cancer (*p* < 0.05) than in low grade bladder cancer and superficial bladder cancer (Figures 9H,I). Kaplan-Meier survival analysis revealed that patients with high expression of *EME1*, *RAD51AP1*, and *RAD54L* genes had a significantly shorter disease-specific survival time than patients with low expression of *EME1*, *RAD51AP1*, and *RAD54* (Figures 9J–M).

### Hub Gene Expression is Associated With Tumor Proliferation and Gene Mutation

**FIGURE 10:**
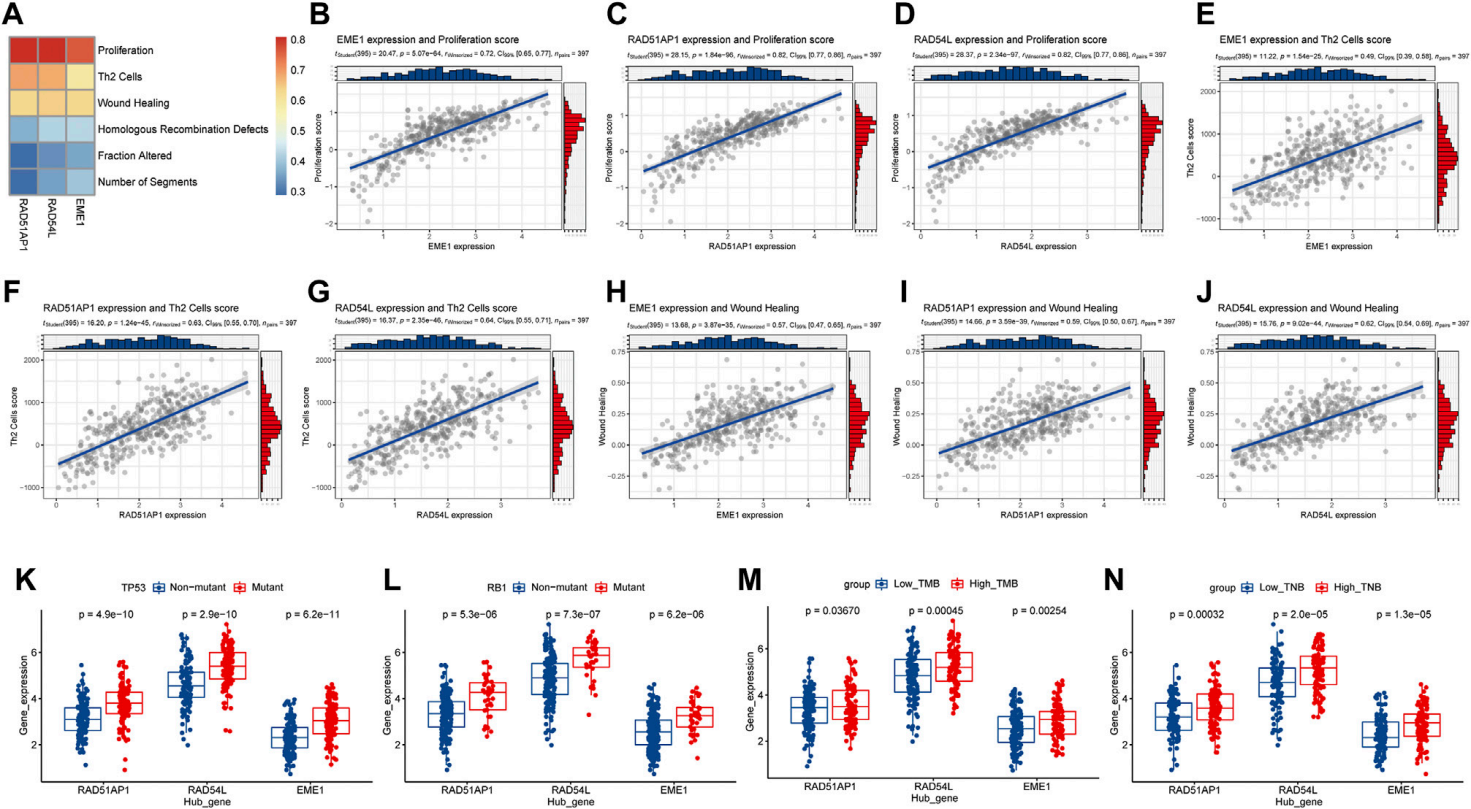
Analysis of the relationship between hub genes in HR and tumor progression and gene mutation. **(A)**, Heatmap of correlation between hub genes and signature scores of core biological functions; **(B–J)**, Correlation diagram between hub gene and the signature scores of core biological functions with moderate correlation; **(K,L)**, Hub gene expression levels in TP53, RB1 gene mutation, and non-mutation samples distribution. **(M,N)**, Distribution of hub gene expression levels in high and low TMB and TNB samples. HR, homologous recombination; TMB, Tumor mutation burden; TNB, tumor neoantigen burden.

The study discovered that the expression levels of hub genes such as *EME1*, *RAD51AP1*, and *RAD54L* were highly positively correlated with Thorsson et al.'s proliferation characteristic data (cor > 0.7, *p* < 0.001), and moderately positively correlated with Th2 cells, Wound Healing, and other characteristic data (cor > 0.4, *p* < 0.001) (Figures 10A–J). The study discovered that *EME1*, *RAD51AP1*, and *RAD54L* were substantially more prevalent in *TP53* and *RB1* mutant samples than in non-mutation samples (*p* < 0.05), as well as in high TMB and TNB samples than in low TMB and TNB samples (Figures 10K–N). The findings indicated that hub genes may enhance tumor growth and are associated with tumor mutation, which may affect immunotherapy.

## Discussion

Immunotherapy was discovered lately, and it now offers new therapeutic choices for individuals with advanced mUC, particularly those who are intolerant to platinum chemotherapy (Witjes et al., 2021). Based on the diversity in treatment among patients, the European Association of Urology recommends utilizing ICIs when the positive number of tumor-infiltrating immune cells evaluated by Ventana SP142 is greater than 5% (Cathomas et al., 2022). However, no attempt has been made to forecast mUC patients’ prognosis using tumor mutation-related characteristics.

The DDR signature genes were scored using ssGSEA, and the results revealed that HR and BER, NHEJ, TLSD, and OTHER were all distinct. The effective group (CR/PR) had much higher HR scores than the ineffective group (SD/PD), whereas the effective group had significantly lower other features than the ineffective group. HR is a process for repairing double-strand DNA breaks that can be used exclusively during the S and G2 stages of the cell cycle due to its reliance on sister chromatids as repair templates (Talens et al., 2017). A recent study indicates that HR defects (HRD) in cancer might result in unrepaired double-strand DNA breaks, fork collapse, genomically rearrangement, and increased tumor aggressiveness in breast and ovarian cancer (Bouwman et al., 2010; Piazza and Heyer, 2019; Vergote et al., 2021). On the other hand, increased HR repair protein production disrupts the HR repair process and promotes gene rearrangement, resulting in genomically instability (Richardson et al., 2004). Furthermore, HR-related genes have been found to be substantially expressed in cancer cells with low HR effectiveness, highlighting that this is a non-functional compensatory strategy in DNA damage repair-deficient malignancies (Pitroda et al., 2014). Patients with a HR deficiency in bladder cancer may benefit more from HRD-targeted platinum medications and PARP inhibitors, and no correlation between their functional activity and bladder cancer has been established (Börcsök et al., 2021). The HR score of the genomically instability sample in the Lund2 subtype was significantly greater than that of the other subtypes, and the genomically instability sample composition in the effective group was significantly higher than that of the ineffective group in the immunotherapy response subgroup. This demonstrates that high HR scores are associated with immunotherapy responses and suggest genomically instability in bladder cancer. Additionally, a variety of other DDR function defects may result in genome and chromosome instability, which may result in DNA leakage from the nucleus to the cytoplasm, activation of cytoplasmic DNA sensors, activation of downstream pathways, including type I interferon response, and ultimately enhanced antitumor immunity, and may result in cancer cells being susceptible to ICIs (Chen et al., 2022). GO functional enrichment analysis showed that HR related modules were closely related to the cell cycle, cell cycle process, and mitotic cell cycle. Excessive cell division is connected with the management of the cell cycle in tumor cells, including cell cycle checkpoints, oncogene-induced replication needs, and mitotic checkpoints (Matthews et al., 2022). Additionally, the heat map demonstrated that samples with a high HR score expressed much more signature genes such as cell cycle, DNA replication, histone, DDR, immunological checkpoint, and CD8+ effector T cells than those with a low HR score. This shows that samples with a high HR score may be more genomically unstable and therefore more susceptible to immunotherapy.

Currently, most immunotherapy prediction models are based on immune cell infiltration. Chen et al. (2021), for example, developed a model based on the CD8+ effector T cell signature’s immune checkpoint signature. Based on the HR gene signature, the study created a 6-gene risk model. In both the training and internal validation groups, patients with a high risk score had a shorter overall survival time than patients with a low risk score, and the ROC curve verified this. This study found risk score and mismatch repair (MMR) to be independent risk factors for immunotherapy patients, proving the risk score’s predictive value. MMR defects result in base pair deletion or mutation at recurrent microsatellite loci, resulting in microsatellite instability (MSI), which is a type of genomic instability (Yoshioka et al., 2021). Defects in DNA double-strand repair have been demonstrated to cause MSI during replication stress in mouse embryonic fibroblasts (Matsuno et al., 2019). Considering HR repair is the primary form of DSB repair, defects in this way may cause MSI as well as MMR defects. Tumor patients with MSI have a substantial number of mutant neoantigens and showed susceptibility to ICIs in cancers of various tissue origins, according to Le et al. (2017), which was consistent with our findings. TNB and CC Reg both are protective factors in their own respect. TNB is more sensitive than TMB in predicting the success of ICIs since it measures the total quantity of neoantigens in tumor cells (Wang et al., 2021). Only a small percentage of mutations can be identified by positive T lymphocytes because not all tumor cell mutations yield neoantigens (Wang and Wang, 2017). The CC reg is also closely related to immunotherapy. By phosphorylating and stabilizing SPOP and boosting its binding to NFB/P65, the cyclin D-CDK4/6 kinase can lower PDL1 expression, which is favorable to immunotherapy (Liu et al., 2022).

The study found RAD51AP1, RAD54L, and EME1 to be hub genes in HR patterns associated with immunotherapy response and tumor progression. RAD51AP1 stands for RAD51-associated protein 1. RAD51AP1 enhances RNA transcript entrance into donor DNA and increases HR by establishing a DR-loop when local transcription is active (Ouyang et al., 2021). Breast cancer, colon cancer, and other cancers express it more. Its defects can slow tumor growth by impairing stem cell self-renewal and increase chemotherapy and radiation sensitivity (Bridges et al., 2020; Bridges et al., 2021). RAD54L is a Rad51 cofactor that participates in HR-related DNA damage repair *via* DNA-dependent double-stranded ATPase(Ceballos and Heyer, 2011). In bladder cancer, the transcription factor E2F1 directly activates RAD54L, regulating DDR, and is linked to poor prognosis (Mun et al., 2020). An endonuclease complex with methyl Methanesulfonate-sensitive UV-Sensitive 81 protein is encoded by EME1. In the absence of CHK1, this endonuclease complex divides poorly replicated chromosomes in mitosis, producing chromosomal instability and tumor development (Calzetta et al., 2020). The finding of these genes in various cancers is related to disease progression. Our study found a substantial correlation between RAD51AP1, RAD54L, and EME1 gene expression and bladder cancer proliferation, Th2, and wound healing scores. The expression levels of hub genes were also higher in the TP53 and RB1 mutant samples than in the non-mutant samples, and in the high TMB and TNB samples than in the low expression levels. This causes uncontrollable cell proliferation and genomically instability due to TP53 and RB1 mutations (Mcclintock, 1938; Frias et al., 2012). This may be the main mechanism acting on tumor mutations.

In a summary, the study discovered that a high HR score was associated with immune response and genomically instability. However, whether the process is driven by HR gene overexpression or by passive non-functional upregulation of HR-related genes caused by DNA damage repair errors remains uncertain, although this has no bearing on the subsequent prediction models’ construction. Additionally, the study identified hub genes related to immunotherapy and tumor growth in HR that will be exploited in future fundamental research.

## Data Availability

The original contributions presented in the study are included in the article/Supplementary Material, further inquiries can be directed to the corresponding authors.

## References

[B1] Babjuk M. Burger M. Zigeuner R. Shariat S. F. van Rhijn B. W. G. Compérat E. (2013). EAU Guidelines on Non-Muscle-Invasive Urothelial Carcinoma of the Bladder: Update 2013. Eur. Urol. 64 (4), 639–653. 10.1016/j.eururo.2013.06.003 23827737

[B2] Barbie D. A. Tamayo P. Boehm J. S. Kim S. Y. Moody S. E. Dunn I. F. (2009). Systematic RNA Interference Reveals that Oncogenic KRAS-Driven Cancers Require TBK1. Nature 462 (7269), 108–112. 10.1038/nature08460 19847166 PMC2783335

[B3] Berglund A. E. Welsh E. A. Eschrich S. A. (2017). Characteristics and Validation Techniques for PCA-Based Gene-Expression Signatures. Int. J. Genomics 2017, 2354564. 10.1155/2017/2354564 28265563 PMC5317117

[B4] Börcsök J. Diossy M. Sztupinszki Z. Prosz A. Tisza V. Spisak S. (2021). Detection of Molecular Signatures of Homologous Recombination Deficiency in Bladder Cancer. Clin. Cancer Res. 27 (13), 3734–3743. 10.1158/1078-0432.CCR-20-5037 33947694 PMC8896908

[B5] Bouwman P. Aly A. Escandell J. M. Pieterse M. Bartkova J. van der Gulden H. (2010). 53BP1 Loss Rescues BRCA1 Deficiency and Is Associated with Triple-Negative and BRCA-Mutated Breast Cancers. Nat. Struct. Mol. Biol. 17 (6), 688–695. 10.1038/nsmb.1831 20453858 PMC2912507

[B6] Bridges A. E. Ramachandran S. Pathania R. Parwal U. Lester A. Rajpurohit P. (2020). RAD51AP1 Deficiency Reduces Tumor Growth by Targeting Stem Cell Self-Renewal. Cancer Res. 80 (18), 3855–3866. 10.1158/0008-5472.CAN-19-3713 32665355 PMC9400129

[B7] Bridges A. E. Ramachandran S. Tamizhmani K. Parwal U. Lester A. Rajpurohit P. (2021). RAD51AP1 Loss Attenuates Colorectal Cancer Stem Cell Renewal and Sensitizes to Chemotherapy. Mol. Cancer Res. 19 (9), 1486–1497. 10.1158/1541-7786.mcr-20-0780 34099522 PMC8612176

[B8] Calzetta N. L. Besteiro M. A. G. Gottifredi V. (2020). Mus81-Eme1-Dependent Aberrant Processing of DNA Replication Intermediates in Mitosis Impairs Genome Integrity. Sci. Adv. 6 (50), eabc8257. 10.1126/sciadv.abc8257 33298441 PMC7725468

[B9] Cathomas R. Lorch A. Bruins H. M. Compérat E. M. Cowan N. C. Efstathiou J. A. (2022). The 2021 Updated European Association of Urology Guidelines on Metastatic Urothelial Carcinoma. Eur. Urol. 81 (1), 95–103. 10.1016/j.eururo.2021.09.026 34742583

[B10] Ceballos S. J. Heyer W.-D. (2011). Functions of the Snf2/Swi2 Family Rad54 Motor Protein in Homologous Recombination. Biochim. Biophys. Acta (Bba) - Gene Regul. Mech. 1809 (9), 509–523. 10.1016/j.bbagrm.2011.06.006 PMC3171615 21704205

[B11] Chan T. A. Yarchoan M. Jaffee E. Swanton C. Quezada S. A. Stenzinger A. (2019). Development of Tumor Mutation Burden as an Immunotherapy Biomarker: Utility for the Oncology Clinic. Ann. Oncol. 30 (1), 44–56. 10.1093/annonc/mdy495 30395155 PMC6336005

[B12] Chen M. Linstra R. Van Vugt M. A. T. M. (2022). Genomic Instability, Inflammatory Signaling and Response to Cancer Immunotherapy. Biochim. Biophys. Acta Rev. Cancer 1877 (1), 188661. 10.1016/j.bbcan.2021.188661 34800547

[B13] Chen X. Xu R. He D. Zhang Y. Chen H. Zhu Y. (2021). CD8+ T Effector and Immune Checkpoint Signatures Predict Prognosis and Responsiveness to Immunotherapy in Bladder Cancer. Oncogene 40 (43), 6223–6234. 10.1038/s41388-021-02019-6 34552192

[B14] Frias C. Pampalona J. Genesca A. Tusell L. (2012). Telomere Dysfunction and Genome Instability. Front. Biosci. 17, 2181–2196. 10.2741/4044 22652771

[B15] Hänzelmann S. Castelo R. Guinney J. (2013). GSVA: Gene Set Variation Analysis for Microarray and RNA-Seq Data. BMC Bioinformatics 14, 7. 10.1186/1471-2105-14-7 23323831 PMC3618321

[B16] Hu Z. Ott P. A. Wu C. J. (2018). Towards Personalized, Tumour-Specific, Therapeutic Vaccines for Cancer. Nat. Rev. Immunol. 18 (3), 168–182. 10.1038/nri.2017.131 29226910 PMC6508552

[B17] Jiang M. Jia K. Wang L. Li W. Chen B. Liu Y. (2021). Alterations of DNA Damage Response Pathway: Biomarker and Therapeutic Strategy for Cancer Immunotherapy. Acta Pharmaceutica Sinica. B. 11 (10), 2983–2994. 10.1016/j.apsb.2021.01.003 34729299 PMC8546664

[B18] Kamoun A. de Reyniès A. Allory Y. Sjödahl G. Robertson A. G. Seiler R. (2020). A Consensus Molecular Classification of Muscle-Invasive Bladder Cancer. Eur. Urol. 77 (4), 420–433. 10.1016/j.eururo.2019.09.006 31563503 PMC7690647

[B19] Kang J. D’Andrea A. D. Kozono D. (2012). A DNA Repair Pathway-Focused Score for Prediction of Outcomes in Ovarian Cancer Treated with Platinum-Based Chemotherapy. J. Natl. Cancer Inst. 104 (9), 670–681. 10.1093/jnci/djs177 22505474 PMC3341307

[B20] Klein A. V. Hambley T. W. (2009). Platinum Drug Distribution in Cancer Cells and Tumors. Chem. Rev. 109 (10), 4911–4920. 10.1021/cr9001066 19711978

[B21] Langfelder P. Horvath S. (2008). WGCNA: An R Package for Weighted Correlation Network Analysis. BMC Bioinformatics 9, 559. 10.1186/1471-2105-9-559 19114008 PMC2631488

[B22] Lausen B. Schumacher M. (1992). Maximally Selected Rank Statistics. Biometrics 48 (1), 73–85. 10.2307/2532740

[B23] Le D. T. Durham J. N. Smith K. N. Wang H. Bartlett B. R. Aulakh L. K. (2017). Mismatch Repair Deficiency Predicts Response of Solid Tumors to PD-1 Blockade. Science 357 (6349), 409–413. 10.1126/science.aan6733 28596308 PMC5576142

[B24] Liu J. Peng Y. Wei W. (2022). Cell Cycle on the Crossroad of Tumorigenesis and Cancer Therapy. Trends Cel Biol. 32 (1), 30–44. 10.1016/j.tcb.2021.07.001 PMC8688170 34304958

[B25] Love M. I. Huber W. Anders S. (2014). Moderated Estimation of Fold Change and Dispersion for RNA-Seq Data with DESeq2. Genome Biol. 15 (12), 550. 10.1186/s13059-014-0550-8 25516281 PMC4302049

[B26] Mariathasan S. Turley S. J. Nickles D. Castiglioni A. Yuen K. Wang Y. (2018). TGFβ Attenuates Tumour Response to PD-L1 Blockade by Contributing to Exclusion of T Cells. Nature 554 (7693), 544–548. 10.1038/nature25501 29443960 PMC6028240

[B27] Massard C. Gordon M. S. Sharma S. Rafii S. Wainberg Z. A. Luke J. (2016). Safety and Efficacy of Durvalumab (MEDI4736), an Anti-Programmed Cell Death Ligand-1 Immune Checkpoint Inhibitor, in Patients with Advanced Urothelial Bladder Cancer. J. Clin. Oncol. 34 (26), 3119–3125. 10.1200/jco.2016.67.9761 27269937 PMC5569690

[B28] Matsuno Y. Atsumi Y. Shimizu A. Katayama K. Fujimori H. Hyodo M. (2019). Replication Stress Triggers Microsatellite Destabilization and Hypermutation Leading to Clonal Expansion *In Vitro* . Nat. Commun. 10 (1), 3925. 10.1038/s41467-019-11760-2 31477700 PMC6718401

[B29] Matthews H. K. Bertoli C. De Bruin R. A. M. (2022). Cell Cycle Control in Cancer. Nat. Rev. Mol. Cel Biol 23 (1), 74–88. 10.1038/s41580-021-00404-3 34508254

[B30] Mcclintock B. (1938). The Production of Homozygous Deficient Tissues with Mutant Characteristics by Means of the Aberrant Mitotic Behavior of Ring-Shaped Chromosomes. Genetics 23 (4), 315–376. 10.1093/genetics/23.4.315 17246891 PMC1209016

[B31] Mun J. Y. Baek S. W. Park W. Y. Kim W. T. Kim S. K. Roh Y. G. (2020). E2F1 Promotes Progression of Bladder Cancer by Modulating RAD54L Involved in Homologous Recombination Repair. Int. J. Mol. Sci. 21 (23), 9025. 10.3390/ijms21239025 PMC7730422 33261027

[B32] Ouyang J. Yadav T. Zhang J.-M. Yang H. Rheinbay E. Guo H. (2021). RNA Transcripts Stimulate Homologous Recombination by Forming DR-Loops. Nature 594 (7862), 283–288. 10.1038/s41586-021-03538-8 33981036 PMC8855348

[B33] Parikh R. B. Adamson B. J. S. Khozin S. Galsky M. D. Baxi S. S. Cohen A. (2019). Association between FDA Label Restriction and Immunotherapy and Chemotherapy Use in Bladder Cancer. JAMA 322 (12), 1209–1211. 10.1001/jama.2019.10650 31550019 PMC6763996

[B34] Park S. Lee H. Lee B. Lee S.-H. Sun J.-M. Park W.-Y. (2019). DNA Damage Response and Repair Pathway Alteration and its Association with Tumor Mutation Burden and Platinum-Based Chemotherapy in SCLC. J. Thorac. Oncol. 14 (9), 1640–1650. 10.1016/j.jtho.2019.05.014 31125737

[B35] Patel V. G. Oh W. K. Galsky M. D. (2020). Treatment of Muscle‐Invasive and Advanced Bladder Cancer in 2020. CA A. Cancer J. Clin. 70 (5), 404–423. 10.3322/caac.21631 32767764

[B36] Piazza A. Heyer W.-D. (2019). Homologous Recombination and the Formation of Complex Genomic Rearrangements. Trends Cel Biol. 29 (2), 135–149. 10.1016/j.tcb.2018.10.006 PMC6402879 30497856

[B37] Pitroda S. P. Pashtan I. M. Logan H. L. Budke B. Darga T. E. Weichselbaum R. R. (2014). DNA Repair Pathway Gene Expression Score Correlates with Repair Proficiency and Tumor Sensitivity to Chemotherapy. Sci. Transl Med. 6 (229), 229ra42. 10.1126/scitranslmed.3008291 PMC4889008 24670686

[B38] Richardson C. Stark J. M. Ommundsen M. Jasin M. (2004). Rad51 Overexpression Promotes Alternative Double-Strand Break Repair Pathways and Genome Instability. Oncogene 23 (2), 546–553. 10.1038/sj.onc.1207098 14724582

[B39] Saginala K. Barsouk A. Aluru J. S. Rawla P. Padala S. A. Barsouk A. (2020). Epidemiology of Bladder Cancer. Med. Sci. (Basel) 8 (1), 15. 10.3390/medsci8010015 PMC7151633 32183076

[B40] Shammas M. A. Shmookler Reis R. J. Koley H. Batchu R. B. Li C. Munshi N. C. (2009). Dysfunctional Homologous Recombination Mediates Genomic Instability and Progression in Myeloma. Blood 113 (10), 2290–2297. 10.1182/blood-2007-05-089193 19050310 PMC2652372

[B41] Simon N. Friedman J. Hastie T. Tibshirani R. (2011). Regularization Paths for Cox's Proportional Hazards Model via Coordinate Descent. J. Stat. Softw. 39 (5), 1–13. 10.18637/jss.v039.i05 PMC4824408 27065756

[B42] Song D. Powles T. Shi L. Zhang L. Ingersoll M. A. Lu Y. J. (2019). Bladder Cancer, a Unique Model to Understand Cancer Immunity and Develop Immunotherapy Approaches. J. Pathol. 249 (2), 151–165. 10.1002/path.5306 31102277 PMC6790662

[B43] Sung H. Ferlay J. Siegel R. L. Laversanne M. Soerjomataram I. Jemal A. (2021). Global Cancer Statistics 2020: GLOBOCAN Estimates of Incidence and Mortality Worldwide for 36 Cancers in 185 Countries. CA A. Cancer J. Clin. 71 (3), 209–249. 10.3322/caac.21660 33538338

[B44] Talens F. Jalving M. Gietema J. A. Van Vugt M. A. (2017). Therapeutic Targeting and Patient Selection for Cancers with Homologous Recombination Defects. Expert Opin. Drug Discov. 12 (6), 565–581. 10.1080/17460441.2017.1322061 28425306

[B45] Thorsson V. Gibbs D. L. Brown S. D. Wolf D. Bortone D. S. Ou Yang T. H. (2018). The Immune Landscape of Cancer. Immunity 48 (4), 812–e14. 10.1016/j.immuni.2018.03.023 29628290 PMC5982584

[B46] Topalian S. L. Taube J. M. Anders R. A. Pardoll D. M. (2016). Mechanism-Driven Biomarkers to Guide Immune Checkpoint Blockade in Cancer Therapy. Nat. Rev. Cancer 16 (5), 275–287. 10.1038/nrc.2016.36 27079802 PMC5381938

[B47] Vergote I. González-Martín A. Ray-Coquard I. Harter P. Colombo N. Pujol P. (2021). European Experts Consensus: BRCA/Homologous Recombination Deficiency Testing in First-Line Ovarian Cancer. Ann. Oncol. 33 (3), 276–287. 10.1016/j.annonc.2021.11.013 34861371

[B48] Wang P. Chen Y. Wang C. (2021). Beyond Tumor Mutation Burden: Tumor Neoantigen Burden as a Biomarker for Immunotherapy and Other Types of Therapy. Front. Oncol. 11, 672677. 10.3389/fonc.2021.672677 33996601 PMC8117238

[B49] Wang R.-F. Wang H. Y. (2017). Immune Targets and Neoantigens for Cancer Immunotherapy and Precision Medicine. Cell Res 27 (1), 11–37. 10.1038/cr.2016.155 28025978 PMC5223235

[B50] Witjes J. A. Bruins H. M. Cathomas R. Compérat E. M. Cowan N. C. Gakis G. (2021). European Association of Urology Guidelines on Muscle-Invasive and Metastatic Bladder Cancer: Summary of the 2020 Guidelines. Eur. Urol. 79 (1), 82–104. 10.1016/j.eururo.2020.03.055 32360052

[B51] Yoshioka K. I. Kusumoto-Matsuo R. Matsuno Y. Ishiai M. (2021). Genomic Instability and Cancer Risk Associated with Erroneous DNA Repair. Int. J. Mol. Sci. 22 (22), 12254. 10.3390/ijms222212254 34830134 PMC8625880

[B52] Zeng D. Ye Z. Shen R. Yu G. Wu J. Xiong Y. (2021). IOBR: Multi-Omics Immuno-Oncology Biological Research to Decode Tumor Microenvironment and Signatures. Front. Immunol. 12, 687975. 10.3389/fimmu.2021.687975 34276676 PMC8283787

[B53] Zhang B. Horvath S. (2005). A General Framework for Weighted Gene Co-Expression Network Analysis. Stat. Appl. Genet. Mol. Biol. 4, 17. 10.2202/1544-6115.1128 16646834

